# Chronic Recurrent Multifocal Osteomyelitis: A Case Report

**Published:** 2011-01-01

**Authors:** E Sadeghi, M R Kadivar, A K Ghadimi moghadam, Gh R Pooladfar, N Sadeghi

**Affiliations:** 1 Department of Pediatrics, Infectious Disease, Nemazee Hospital, Shiraz University of Medical Sciences, Shiraz, Iran; 2 Professor Alborzi Clinical Microbiology Research Center, Nemazee Hospital, Shiraz University of Medical Sciences, Shiraz, Iran; 3 Department of Pediatrics, Urmia University of Medical Sciences, Urmia, Iran

**Keywords:** CRMO, Chronic osteomyelitis, NSAID, Recurrent osteomyelitis

## Abstract

Chronic recurrent multifocal osteomyelitis (CRMO) is a rare inflammatory bone disease. It is a diagnosis of exclusion based on the clinical, radiological and pathological criteria. The awareness of the corresponding feature can help avoid of unnecessary diagnostic procedures and prolonged antibiotic therapy. We present a case of 4.5 year old girl, diagnosed with CRMO who were followed for 6 months as a case of chronic bacterial osteomyelitis and received long course of antibiotic therapy.

## Introduction

Chronic recurrent multifocal osteomyelitis (CRMO) is a rare inflammatory bone disease which was first described in 1972 as ‘‘an unusual form of multifocal bone lesions with subacute and chronic symmetrical osteomyelitis” [1][2][3]. Since then, more than 200 cases have been reported [2][4]. CRMO is a diagnosis of exclusion based on the clinical, radiological and pathological criteria [2][5]. Local pains and swelling with gradual onset, multifocal lesions displaying characteristic radiological features, failure to cultivate an infectious organism, improvement by anti-inflammatory drugs, and a protracted course for years with recurrent exacerbations are the characteristic features of CRMO [1][2][5][6].

The awareness of the disease and its features can help avoid unnecessary diagnostic procedures and prolonged antibiotic therapy. We present a case of 4.5 year old girl, diagnosed with CRMO who were followed for 6 months as a case of chronic bacterial osteomyelitis and received long course of antibiotic therapy.

## Case Report

In March 2008, a 4.5 year old female child who presented with sudden onset of left leg pain and limping over the last 10 days was admitted with impression of osteomyelitis. She did not have any history of trauma. Her appetite and weight were normal for her age. There was no history of fever. She had no other skeletal or joint complaints. She did not suffer from any major medical problems, either. She was afebrile and her general condition was goodl. A mild swelling, tenderness, hotness and pain on motion were detected in her right leg, and in otherwise general physical exam including skin and neurological exam she was normal. There was no family history of skeletal problems.

**Fig. 1:**
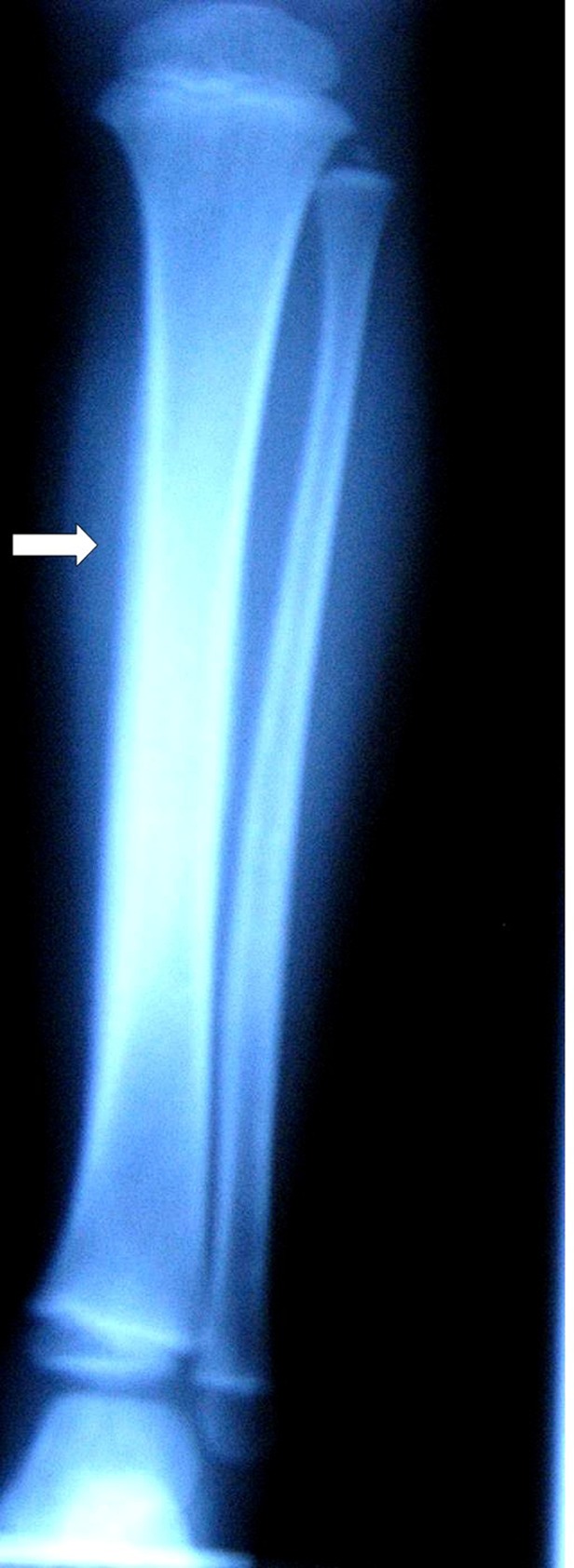
Right leg simple X-ray shows periosteal elevation.

**Fig. 2:**
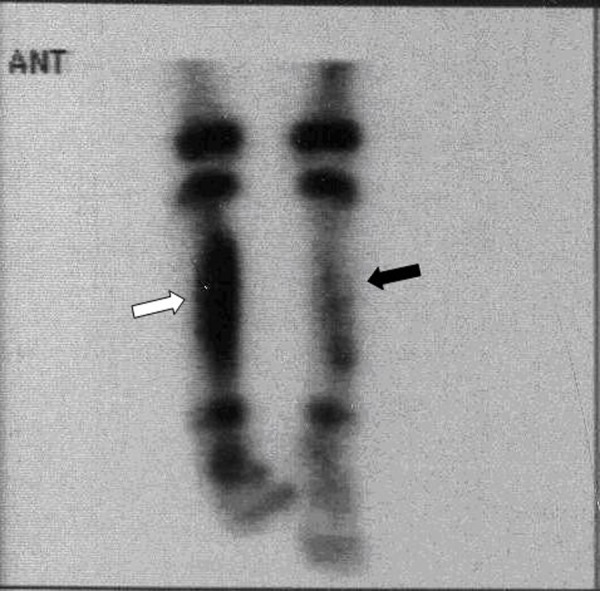
Bone scintigraphy three hours after i.v. injection Tc99m shows significantly increased uptake in right tibia (white arrow) and slightly increased uptake in the left tibial bone (black arrow).

Full blood count, C-reactive protein (CRP) and erythrocyte sedimentation rate (ESR) were normal. Blood culture was negative for pyogenic organisms. X-ray of her left leg revealed periosteal reaction of diaphysis of left tibia with soft tissue swelling (Figure 1). Isotope bone scan revealed increased uptake on the late films along the left tibial bone (Figure 2). Antibiotic therapy was started and open biopsy and curettage was performed. Cultures of bone specimen did not yield any organisms. Pathologic evaluation of the samples, reported the inflammatory changes and fibrosis which were consistent with subacute and chronic inflammation and there was no evidence of neoplasia. After 10 days, the clinical signs and symptoms of the patient resolved. The patient was followed in out-patient clinic as a case of chronic osteomyelitis and received oral clindamycin.

**Fig. 3:**
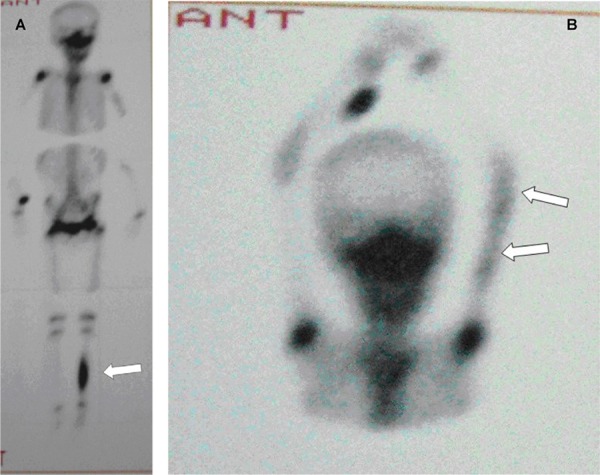
A. Bone scintigraphy three hours after i.v. injection Tc99m show active pathology in the left tibia and, B. in entire length of the left humerus.

**Fig. 4:**
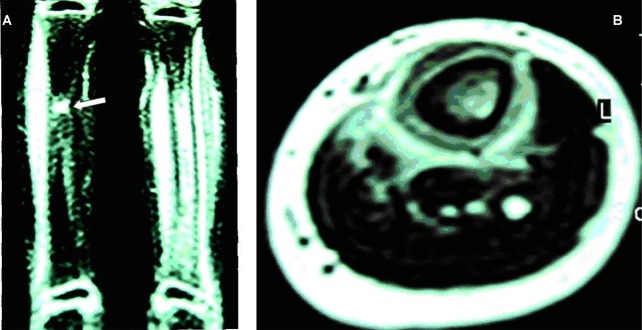
A. Coronal STIR MRI demonstrated tibial marrow edema in the shaft and surrounding soft tissue, also note minor edema of right tibia were in favor of osteomyelitis. The arrow shows site of previous bone biopsy of right tibia. B. Axial T1 weighted image of left leg reveal decrease in signal intensity in the tibial marrow, periosteal elevation and edema of soft tissue.

In September 2008, she was readmitted due to limping and pain on the left leg and clindamycin continued intravenously. She was afebrile. A tenderness on the left leg and mild tenderness in the left arm were detected in physical examination. Full blood count, C-reactive protein (CRP) and erythrocyte sedimentation rate (ESR) were again normal. Blood culture was negative. Isotope bone scan revealed active bony pathology in nearly all parts of the left humerus and left tibial shaft (Figure 3). MRI of the left leg revealed a significant increase in signal intensity on STIR sequence at the shaft of the left tibial bone and extension of signal change into distal metaphysis of bone and evidence of significant inflammatory change in deep soft tissue structures around the tibial bone. MRI of right tibial bone showed an increase in signal intensity on STIR sequence at the mid shaft, however, there was no evidence of inflammatory change in the surrounding soft tissue structures (Figure 4). There was no evidence of collection or abscess formation in the soft tissue structure of the right leg. Bone biopsy of the left tibia did not reveal any organism and pathologic findings were compatible with chronic inflammation. Acid fast and KoH staining were negative. Immunological work ups including immunoglobulin levels, CH50, nitroblue tetrazolium test and flow cytometery for white blood cells were normal. Serology for HIV was inconclusive. Based on the history and physical examination findings, the patient was diagnosed as a case of chronic recurrent multifocal osteomyelitis (CRMO). Accordingly, clindamycin was stopped and non-steroidal anti inflammatory medication (ibuprofen) started. She did not exhibit recurrence of pain in any her limbs and no sequella was developed during the 18 months follow up.

## Discussion

Chronic recurrent multifocal osteomyelitis is a rare variant of osteomyelitis, accounting for 2% to 5% of cases of osteomyelitis [7]. In the second admission, the patient was diagnosed as a case of CRMO according to the clinical, radiological and pathological criteria [2][5]. Indeed, CRMO is a diagnosis of exclusion based on the following criteria: a) bone lesions with a radiographic picture suggesting subacute or chronic osteomyelitis; b) an unusual location of lesions when compared with infectious osteomyelitis with a frequent multifocality; c) no abscess formation, fistula, or sequestra; d) lack of a causative organism; e) nonspecific histopathological and laboratory findings compatible with subacute or chronic osteomyelitis; f) a characteristic prolonged, fluctuating course with recurrent episodes of pain occurring and g) occasional accompanying skin diseases, most frequent of which pustulosis palmoplantaris (PPP) and less commonly acne, psoriasis vulgaris, and pyoderma gangrenosum [2]. Characteristic recurrent episodes of pain, multifocal location of lesions (3 total bony lesions), absence of fistula, sequestra or abscess formation, lack of a causative organism, histopathological findings compatible with subacute or chronic osteomyelitis, were characteristic feartures of CRMO in our patient [3][8][9][10][11][12].

Other causes of osteomyelitis such as infectious osteomyelitis, juvenile idiopathic arthritis, Ewing's sarcoma, metastatic neuroblastoma, hematolymphoid malignancy, Langerhans cell histiocytosis and chronic infection, notably tuberculosis were excluded [2][5]. Pathologic investigation plays a major role in ruling out other diagnoses. In a considerable number of patients, diagnostic imaging alone can not rule out malignancy; therefore, biopsy should be considered, especially because it is often difficult to make a definite distinction between oncologic bone lesions and those associated with CRMO [13].

Most clinical features of our patient are compatible with previous reports. CRMO mainly affects the girls and it occurs predominantly in children and adolescents as in our patient [3][10][14]. The average age of the patients was reported to be about 10 years and the youngest patient has been 17 months old [8][15][17]. Lower limb bones are the most often affected sites (55%) [1]. Bone pain as an initial symptom was reported in all the 40 patients and fever in only nine patients [3]. As in the present report, bacteriologic investigation of the biopsy specimen is often negative, suggesting that the inflammatory process might have become independent of the initial bacterial infection [6][8][10][11].

Some common features of CRMO were absent in our case. Brown et al. reported inflammatory markers increased in 65% of the 11 patient's series [18] and in Catalano-pons et al. reported 40 CRMO series, 68% of the patients had increased inflammatory markers,[3] however, in our case inflammatory markers were normal. Typical locations of CRMO are the metaphyses of the long bones [3][8][10][19]. The site of involvement in our patient was diaphysis with extension to metaphysis.

In general, treatment options include watchful waiting for spontaneous remission [20] although treatments with NSAIDS, [1][2][6][9][10][11][13][15][21] corticosteroids, [2][9][11] pamidronate,[9][10][22] sulfasalazine,[1][2][13] Gamma INF,[2][6][9][10][23] and INF alfa blockage, [9][10][19] have been reported with some success. Our patient had a good response to NSAIDs without recurrence during 18 month follow up. Indeed, NSAIDS are the treatment of choice for CRMO [1][7][9][10]. NSAIDs can induce remission in up to 85% of the patients with CRMO. Remission is defined as no pain, a decrease in inflammatory markers back to normal values, and no radiological progression [10]. The treatment goals for CRMO are maintaining normal bone growth and function of the adjacent joint. Antibiotic treatment is considered ineffective [1][6][10][11]. A rapid course of corticosteroids is recommended in refractory cases [1][10][11][22].

It is concluded that the awareness of the characteristic features of CRMO could help avoiding unnecessary diagnostic procedures and prolonged antibiotic therapy.
